# Chemical Composition and Bioactivities of the Essential Oil from *Etlingera yunnanensis* against Two Stored Product Insects

**DOI:** 10.3390/molecules200915735

**Published:** 2015-08-28

**Authors:** Shan-Shan Guo, Chun-Xue You, Jun-Yu Liang, Wen-Juan Zhang, Zhu-Feng Geng, Cheng-Fang Wang, Shu-Shan Du, Ning Lei

**Affiliations:** 1 Beijing Key Laboratory of Traditional Chinese Medicine Protection and Utilization, Beijing Normal University, No.19 Xinjiekouwai Street, Beijing 100875, China; E-Mails: guoshanshan@ mail.bnu.edu.cn (S.-S.G.); youchunxue@mail.bnu.edu.cn (C.-X.Y.); liangjunyu@nwnu.edu.cn (J.-Y.L.); zwj0729@mail.bnu.edu.cn (W.-J.Z.); gengzhufeng@bnu.edu.cn (Z.-F.G.); wangchengfang@mail.bnu.edu.cn (C.-F.W.); 2 Analytical and Testing Center, Beijing Normal University, Beijing 100875, China; 3 Department of Pharmacy General Hospital of Second Artillery, PLA, Haidian District, Beijing 100088, China

**Keywords:** *Etlingera yunnanensis*, contact activity, repellency, composition, stored product insects

## Abstract

The chemical composition of the essential oil of *Etlingera yunnanensis* rhizomes and its contact and repellent activities against *Tribolium castaneum* (Herbst) and *Liposcelis bostrychophila* (Badonnel) were investigated. The essential oil obtained from *E. yunnanensis* rhizomes with hydrodistillation was performed by gas chromatography-flame ionization detection and gas chromatography-mass spectrometry. The main components of the essential oil were identified to be estragole (65.2%), β-caryophyllene (6.4%), 1,8-cineole (6.4%), limonene (5.2%), and α-pinene (2.4%). It was found that the essential oil of *E. yunnanensis* rhizomes possessed contact toxicity against *T. castaneum* and *L. bostrychophila* (LD_50_ = 23.33 μg/adult and LD_50_ = 47.38 μg/cm^2^, respectively). Estragole, 1,8-cineole, and limonene exhibited stronger contact toxicity (LD_50_ values of 20.41, 18.86, and 13.40 μg/adult, respectively) than β-caryophyllene (LD_50_ = 41.72 μg/adult) against *T. castaneum* adults. Estragole possessed stronger contact toxicity (LD_50_ = 30.22 µg/cm^2^) than β-caryophyllene, 1,8-cineole, and limonene (LD_50_ values of 74.11, 321.20, and 239.62 μg/adult, respectively) against *L. bostrychophila* adults. Repellency of the crude oil was also evaluated. The essential oil and constituents possessed strong repellent activity against *T*. *castaneum* adults. The four individual constituents showed weaker repellent activity than the essential oil against *L. bostrychophila* adults. The results indicated that the essential oil of *E. yunnanensis* rhizomes and the individual constituents had the potential to be developed as a natural insecticide and repellent for the control of *T. castaneum* and *L. bostrychophila.*

## 1. Introduction

The widespread extensive use of synthetic insecticides has triggered many negative consequences (*i.e.*, insecticide resistance, toxicity to mammals and other non-target animals, residue problems, environmental pollution) [1,2]. Risks associated with the use of synthetic insecticides have led to the growth of an environmental movement seeking sustainable alternatives in pest control [3]. Therefore, increasing attention is being given to natural products. Plants can provide potential alternatives to the currently used insecticides that seem to cause insecticide resistance and environmental and human health concerns because they constitute a rich source of bioactive chemicals, such as terpenoids, alkaloids, and avonoids, against insects, and they have evolved strategies to interact with other organisms for self-defense [4]. Many essential oils and their constituent compounds from plants have been evaluated for repellency and insecticidal activity against stored product insects and some of them are quite promising in the development of natural repellents or insecticides [5,6,7,8,9].

In ancient China, extracts of many medicinal herbs and spices were used to control grain storage insects and pests [10]. Moreover, a traditional Chinese medicinal material conservation method called antagonistic storage has also been used for medicinal materials that have special volatile odors to prevent the insects in other medicinal materials [11]. However, the gap between unsubstantiated traditional use and experimentally substantiated potential should be bridged. Thus, based on the experimental evidence, this traditional method should be developed and inherited.

In order to develop this traditional method of prevention and control of storage pests, we have established a screening program and focus on the volatile substances due to their major role in the antagonistic storage process. During this screening process, the essential oil of *Etlingera yunnanensis* (T.L. Wu and S.J. Chen) R.M. Smith [12] was found to possess insecticidal activity against the red flour beetle, *Tribolium castaneum* Herbst, and the booklouse, *Liposcelis bostrychophila* Badonnel. The red flour beetle is one of the most widespread and destructive insect pests of stored cereals, and can be found where grains or other dried foods are stored [13]. Infestations not only cause significant losses due to the consumption of grains but also result in elevated temperature and moisture conditions, which lead to accelerated growth of molds, including toxigenic species [14]. Booklice have a worldwide distribution infesting domestic premises, raw material stores, manufacturing factories, and historical documents in museums. Additionally, new evidence indicates that psocids are perhaps the most important emerging pests in stored grains and related commodities due to their small size, and resistance to chemicals [15].

*Etlingera*, a genus in Zingiberaceae, has only two species, *Etlingera littoralis* and *Etlingera yunnanensis*, distributed in China [16]. *E. yunnanensis* is a perennial herb, and it has an attractive inflorescence-like chrysanthemum. When it is frayed, it releases an anis-like odor [12]. It is commonly used in Dai medicine and is called “Maniangbu” in a different clinical usage, *i.e.*, for diarrhea and sunstroke [17]. A literature survey has shown that there is neither a report on the chemical composition of *E. yunnanensis* rhizomes essential oil nor a report on its insecticidal and repellent activity. Therefore, we decided to evaluate the contact and repellent activity of the essential oil of *E. yunnanensis* rhizomes and its main compounds against two stored product insects.

## 2. Results and Discussion

### 2.1. Chemical Composition of the Essential Oil

**Figure 1:**
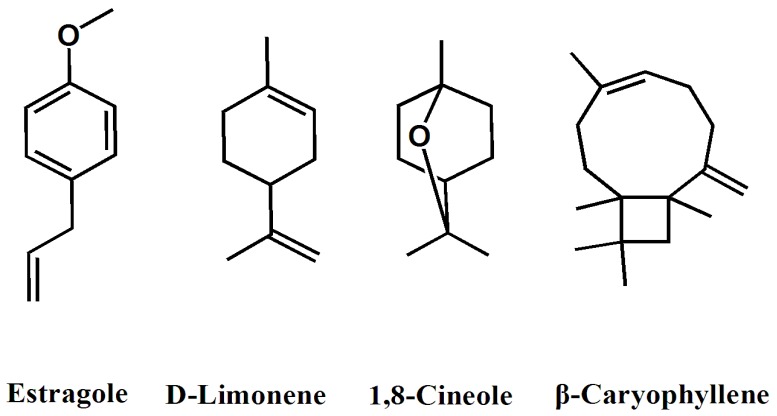
The structure of the main components in essential oil.

The yield of yellow essential oil from *E. yunnanensis* rhizomes was 0.14% (*v*/*w*) and the density of the essential oil was determined to be 0.97 g/mL. The chemical composition of the essential oil was summarized in Table 1. A total of 12 compositions of the essential oil were identified. The principal components of the essential oil were estragole (65.2%), β-caryophyllene (6.4%), 1,8-cineole (6.4%), and limonene (5.2%) (Figure 1).

**Table 1:** Chemical composition of *Etlingera yunnanensis* essential oil.

Peak No.	Compound	RI *	Area %
1	α-Pinene	932	2.4
2	Camphene	943	0.1
3	Limonene	1029	5.2
4	1,8-Cineole	1032	6.4
5	Camphor	1120	1.0
6	α-Terpineol	1189	0.6
7	Estragole	1197	65.2
8	Dodecane	1203	0.1
9	Isobornyl acetate	1286	2.7
10	β-Caryophyllene	1420	6.4
11	α-Caryophyllene	1454	0.7
12	α-Farnesene	1489	0.1
	Monoterpenoids		15.7
	Sesquiterpenes		7.2
	Phenylpropanoid		65.2
	Others		2.8
	Total		90.9

* RI, retention index as determined on a HP-5MS column using the homologous series of *n*-alkanes (C_5_–C_36_).

The essential oil from *E. yunnanensis* rhizomes consisted mainly of phenylpropanoid (estragole), which accounted for 65.2%. Estragole (Figure 1) is a phenylpropene, which consists of a benzene ring substituted with a methoxy group and a propenyl group. It is an isomer of anethole, differing with respect to the location of the double bond [18]. Its name derives from “estragon”, the French and German word for tarragon (*Artemesia dracunculus*), a herb to which it gives its anis-like odor [19]. Furthermore, it is also the primary constituent of the essential oil of anise, fennel [20], basil [21,22], *Croton zehntneri* [23], *Clausena anisata* [24], *Zanthoxylum schinifolium* [25,26], *Lonicera japonica* [27], and so on.

According to the previous paper about the essential oil from other organs of *E. yunnanensis*, the main components of the leaves of *E. yunnanensis* are comprised mainly of germacrene D (19.2%), β-pinene (11.6%), and α-amorphene (11.2%), while the stems are rich in β-pinene (23.7%), 1,8-cineole (11.0%), and α-pinene (9.6%). The major components of the root oil of *E. yunnanensis* were β-pinene (31.9%), α-pinene (13.7%), and 1,8-cineole (9.4%) [28]. Compared with other parts of *E. yunnanensis*, the rhizome oil also had some common compounds, including 1,8-cineole and α-pinene. However, some compounds like germacrene D and α-amorphene were not detected in the essential oil of its rhizomes. In addition, compared with the other two plants from *Etlingera*, their essential oils are also characterized by high levels of phenylpropanoids, especially in the rhizomes [29]. These differences in chemical composition and content between the essential oils of leaves, stems, roots, and rhizomes could be due to the different effects of environmental factors (such as sunlight, water, and soil) on the aerial parts and underground parts, or may result from different metabolic pathways in the plant [30]. Thus, further studies on plant cultivation and essential oil standardization are necessary.

### 2.2. Contact Toxicity

**Figure 2:**
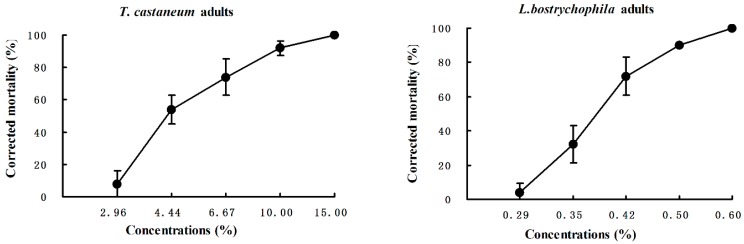
Contact toxicity of *Etlingera yunnanensis* rhizome essential oil against *Tribolium castaneum* and *Liposcelis bostrychophila.*

The essential oil of *E. yunnanensis* rhizomes showed contact toxicity against *T. castaneum* adults with a LD_50_ value of 23.33 μg/adult (Table 2). When compared with the positive control pyrethrum, the essential oil demonstrated 89.62 times less toxic activity against *T. castaneum* adults. *E. yunnanensis* essential oil also possessed contact toxicity (LD_50_ = 47.38 µg/cm^2^) against the booklice. When compared with the positive control pyrethrum, the essential oil showed 2.53 times less toxic activity against *T. castaneum* adults (Table 2). However, compared with the other essential oils in the literature, the essential oil of *E. yunnanensis* possessed stronger contact toxicity against *L. bostrychophila* than, for example, essential oils of *Lonicera japonica* (LD_50_ = 64.04 µg/cm^2^) [27], *Litsea cubeba* (LD_50_ = 71.56 µg/cm^2^) [31], *Foeniculum vulgare* (LD_50_ = 90.36 µg/cm^2^) [5], *Acorus calamus* (LD_50_ = 100.21 µg/cm^2^) [32], *Curcuma wenyujin* (LD_50_ = 208.85 µg/cm^2^) [33], and *Artemisia rupestris* (LD_50_ = 418.48 µg/cm^2^) [34]. The concentration-response curves for the two toxicity assays against *Tribolium castaneum* and *Liposcelis bostrychophila* were showed in Figure 2.

**Table 2:** Contact toxicity of *Etlingera yunnanensis* rhizomes essential oil and its constituents against *Tribolium castaneum* (TC) and *Liposcelis bostrychophila* (LB).

Insects	Treatments ***	LD_50_ (µg/adult; µg/cm^2^)	95% FL (µg/adult; µg/cm^2^)	Slope ± SE	Chi Square (χ^2^)	*p*-Value
TC	Essential oil	23.33	20.09–25.27	5.07 ± 0.57	13.29	0.946
	Estragole	20.41	18.95–22.36	5.18 ± 0.53	15.64	0.87
	β-Caryophyllene	41.72	37.94–45.52	5.28 ± 0.54	18.58	0.725
	1,8-Cineole	18.86	17.51–20.65	5.04 ± 0.51	18.00	0.757
	Limonene	13.40	11.72–15.49	3.23 ± 0.41	9.96	0.992
	Pyrethrins *	0.26	0.22–0.30	3.34 ± 0.32	13.11	0.950
LB	Essential oil	47.38	45.66–49.11	14.09 ± 1.51	10.02	0.991
	Estragole	30.22	28.13–32.49	8.04 ± 0.96	6.25	0.995
	β-Caryophyllene	74.11	71.26–77.75	10.40 ± 1.08	20.11	0.635
	1,8-Cineole	321.20	302.38–333.90	12.49 ± 1.31	11.37	0.979
	Limonene	259.62	238.13–283.68	5.56 ± 0.57	16.10	0.851
	Pyrethrins **	18.72	17.60–19.92	2.98 ± 0.40	10.56	0.987

* Data from You *et al.* [35]; ** data from Yang *et al.* [31]. *** The mortality of the control (acetone) was 0 μg/adult for TC and 0 µg/cm^2^ for LB.

The main constituent compounds estragole, 1,8-cineole, and limonene exhibited stronger contact toxicity against the red flour beetles, with LD_50_ values of 20.41, 18.86, and 13.40 μg/adult, respectively than the essential oil (Table 2), while β-caryophyllene exhibited weaker toxicity against *T. castaneum* adults (LD_50_ = 41.72 μg/adult). However, only estragole possessed stronger contact toxicity (LD_50_ = 30.22 µg/cm^2^) than the essential oil against the booklice. The previous reports using a similar bioassay method also mentioned the contact activity of the four constituents and the results were about the same [26,27,31,35,36]. Therefore, as the uppermost constituent in the essential oil of *E. yunnanensis*, estragole is one of the contributors to the contact activity of the essential oil. Furthermore, estragole had been shown to exhibit contact toxicity against several stored product insects such as *Sitophilus zeamais* [22,25], *Sitophilus oryza*, *Callosobruchus chinensis*, and *Lasioderma serricorne* [37]. Moreover, some modes of action of estragole were also found. From electrophysiological experiments, Huignard *et al.* observed that estragole specifically induces a reduction of posthyperpolarization [38]. Additionally, a prior study about acetylcholinesterase demonstrated that estragole, as well as *s*-carvone and camphor, produced a mixed inhibition for this enzyme, binding either to the free enzyme or to the enzyme-substrate complex but linking to a site different from the active site where the substrate binds [39]. Thus, the insecticidal activity of the essential oil of *E. yunnanensis* and estragole was quiet promising.

On the other hand, as the uppermost constituent in the essential oil of *E. yunnanensis* rhizomes, estragole has been demonstrated to be genotoxic and carcinogenic. Consequently, reductions in exposure and restrictions in use levels as a flavoring substance have been recommended by the Scientific Committee on Food [40]. European Union regulations on flavorings have also established maximum levels for estragole in certain compound foods resulting from the use of food ingredients in which it naturally occurs [41]. Moreover, based on the adverse effects and risk assessments, the security doses of some main constituents must be studied further [42]. This point is best illustrated with the example of 1,8-cineole. This constituent can be used internally as a flavoring and medicinal ingredient at very low doses, but it is toxic if ingested in greater than normal doses. Although this medicinal herb is safe for human consumption because it has been used as a folk medicinal herb for hundreds of years, no experimental data about its safety is available so far. Essential oils are indeed natural, but this origin does not imply that they are safe. In terms of toxicokinetics and ecotoxicology, essential oils also have some potential problems [3]. The latter observations imply that use of essential oils can be not safe for beneficial insects as natural enemies and pollinators. Thus, to develop a practical application for the essential oil and estragole as novel insecticides, further research on the safety and effectiveness of the essential oil for humans is needed.

### 2.3. Repellency

**Figure 3:**
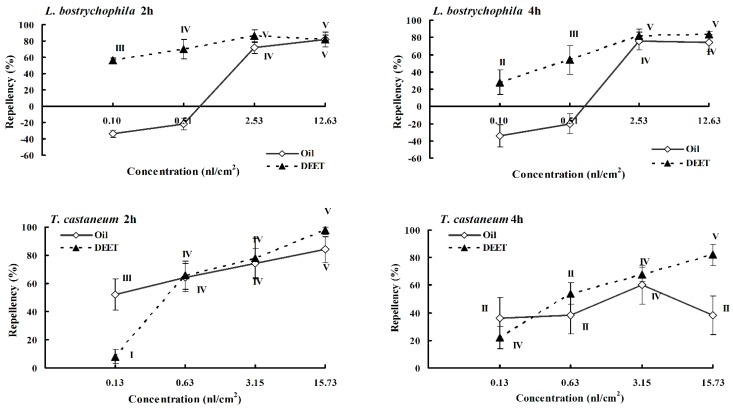
Percentage repellency of DEET and the essential oil from *Etlingera yunnanensis* rhizomes against *Tribolium castaneum* and *Liposcelis bostrychophila* at 2 h and 4 h after exposure. The averages were assigned to different classes (0 to V) using percentage repellency. Vertical error bars indicated standard error of mean.

The results of repellency assays for the essential oil against the two species of stored product insects were presented in Table 3 and Table 4 and Figure 3. Data showed that at tested concentrations, the essential oil possessed strong repellency against *T*. *castaneum* adults. At the lowest assayed concentration (0.13 nL/cm^2^), the essential oil still showed 36% repellency against the beetles at 4 h after exposure. However, the essential oil only exhibited strong repellency against *L. bostrychophila* at the dose of 12.63 nL/cm^2^ and 2.53 nL/cm^2^. At the other concentrations, the two compounds showed some insect-attractant properties.

The four main constituents showed different repellent activity against the two stored product insects (Table 3 and Table 4). At the concentration of 15.73 nL/cm^2^, estragole, β-caryophyllene, and limonene showed strong (Class V) repellency (94%, 80%, and 88%) against *T*. *castaneum* adults at 2 h after exposure (Table 3), while 1,8-Cineole exhibited moderate (Class III) repellency (50%) against the red flour beetles. However, estragole, β-caryophyllene, 1,8-cineole, and limonene all showed a lower level of repellency (54%, 36%, 38%, and 64%, respectively) against *L. bostrychophila* at the dose of 12.63 nL/cm^2^ after 2 h treatment (Table 4).

In previous reports, estragole, limonene, 1,8-cineole, and β-caryophyllene all showed repellent activity against the red flour beetles, and Yang *et al.* reported the repellency of limonene against booklice [31,35,36]. Many essential oils and their constituents were evaluated for repellency against insects as well [43]. The data and literature survey has indicated that the bioactivity properties of essential oils may be related to the synergistic effects of their diverse major and minor components [35]. This paper reported that the essential oil of *E. yunnanensis* had contact and repellent activities to red flour beetles and booklice for the first time. These findings, considered together, suggest that the essential oil showed potential for development as a natural insecticide/repellent for stored products.

**Table 3:** Percentage repellency (PR) after two exposure times for the essential oil and its constituents against *Tribolium castaneum* (TC) adults ^a^.

Insect	Treatment	2 h/4 h (nL/cm^2^)			
		15.73	3.15	0.63	0.13
TC	Essential oil	84 ± 9ab; 38 ± 14a	74 ± 11b; 60 ± 14a	64 ± 10a; 38 ± 13a	52 ± 11bc; 36 ± 15ab
	Estragole	94 ± 6b; 82 ± 5b	88 ± 5b; 68 ± 15a	78 ± 13a; 64 ± 15a	58 ± 13bc; 48 ± 16ab
	β-Caryophyllene	80 ± 8ab; 98 ± 3b	68 ± 8ab; 90 ± 5a	38 ± 12a; 36 ± 13a	36 ± 14ab; 34 ± 13ab
	1,8-Cineole	50 ± 13a; 74 ± 16ab	28 ± 14a; 54 ± 15a	42 ± 16a; 38 ± 12a	28 ± 14ab; 40 ± 10ab
	Limonene	88 ± 14b; 94 ± 8b	70 ± 9b; 56 ± 16a	76 ± 11a; 56 ± 14a	78 ± 10c; 70 ± 12b
	DEET	98 ± 3b; 82 ± 8b	78 ± 14b; 68 ± 5a	66 ± 10a; 54 ± 8a	8 ± 5a; 22 ± 8a

^a^ Means in the same column followed by the same letters do not differ significantly (*p* > 0.05) in ANOVA and Tukey’s tests. PR was subjected to an arcsine square-root transformation before ANOVA and Tukey’s tests.

**Table 4:** Percentage repellency (PR) after two exposure times for the essential oil and its constituents against *Liposcelis bostrychophila* (LB) ^a^.

Insect	Treatment	2 h/4 h nL/cm^2^			
		12.63	2.53	0.51	0.10
LB	Essential oil	82 ± 9ab; 74 ± 10a	72 ± 7a; 76 ± 10a	−22 ± 7a; −20 ± 12a	-34 ± 4bc; −34 ± 13ab
	Estragole	−54 ± 9b; −40 ± 3b	76 ± 6a; 38 ± 16a	0 ± 16a; 20 ± 15a	22 ± 11bc; −30 ± 12ab
	β-Caryophyllene	36 ± 8ab; 24 ± 18b	−24 ± 8ab; 32 ± 14a	24 ± 16a; 42 ± 14a	24 ± 15ab; 32 ± 16ab
	1,8-Cineole	38 ± 14a; 28 ± 12ab	12 ± 8a; 6 ± 14a	−10 ± 10a; 10 ± 14a	−16 ± 11ab; 14 ± 11ab
	Limonene	64 ± 7b; 52 ± 10b	30 ± 10b; 16 ± 11a	24 ± 15a; 20 ± 6a	36 ± 13c; 18 ± 12b
	DEET	82 ± 5b; 84 ± 3b	86 ± 8b; 82 ± 8a	70 ± 12a; 54 ± 17a	56 ± 3a; 28 ± 14a

^a^ Means in the same column followed by the same letters do not differ significantly (*p* > 0.05) in ANOVA and Tukey’s tests. PR was subjected to an arcsine square-root transformation before ANOVA and Tukey’s tests.

## 3. Experimental Section

### 3.1. Plant Material and Extractions

Rhizomes of *Etlingera yunnanensis* were collected in June 2013 from Yunnan Province, China. The species was identified according to the voucher specimen (BNU-CMH-Dushushan-2013-06-15-015) deposited at the Herbarium of College of Resources Science and Technology, Beijing Normal University. Rhizomes (1.4 kg) of *E. yunnanensis* were subjected to hydrodistillation by a modified Clevenger-type apparatus for 6 h and then extracted with *n*-hexane. After extraction, water in the essential oil was removed by anhydrous sodium sulphate. The essential oil was stored in an airtight container in a refrigerator at 4 °C.

### 3.2. Insects

The *T. castaneum* and *L. bostrychophila* were obtained from laboratory cultures maintained for the last two years in incubators at 29 ± 1 °C and 70%–80% relative humidity in the dark. Adult *T. castaneum* insects were reared in glass containers (0.5 L) containing at 12%–13% moisture content on whole wheat mixed with yeast (wheatfeed/yeast, 10:1, *w*/*w*) while booklice were reared on a 10:1:1 mixture, by mass, of flour, milk powder, and active yeast. Adults used in all the experiments were about 1–2 weeks old. All the containers housing booklice used in experiments were made escape-proof with a coating of polyterafluoroethylene (Sino-rich^®^, Beijing Sino-rich Tech Co., Ltd., Xuanwu District, Beijing, China).

### 3.3. Gas Chromatography and Mass Spectrometry (GC-MS)

GC-MS analysis was performed on a Thermo Finnigan Trace DSQ instrument equipped with a flame ionization detector and an HP-5MS (30 m × 0.25 mm × 0.25 μm) capillary column. The column temperature was programmed at 50 °C for 2 min, then increased at 2 °C/min to the temperature of 150 °C and held for 2 min, and then increased at 10 °C/min until the final temperature of 250 °C was reached, where it was held for 5 min. The injector temperature was maintained at 250 °C and the volume injected was 0.1 mL of 1% solution (diluted in acetone). The carrier gas was helium at a flow rate of 1.0 mL/min. Spectra were scanned from 50 to 550 *m*/*z*. Most constituents were identified by comparison of their retention indices with those reported in the literatures. The retention indices were determined in relation to a homologous series of *n*-alkanes (C_5_–C_36_) under the same operating conditions. GC retention time and their mass spectra that were stored in NIST 05 and Wiley 275 libraries or from literature were used for identifying the essential oil components [44]. Relative percentages of the individual components of the essential oil were obtained by averaging the GC peak area % reports.

### 3.4. Contact Toxicity

The contact toxicity of the essential oil and the individual compounds against *T. castaneum* adults was measured as described [12]. The four individual compounds (estragole, β-caryophyllene, 1,8-cineole, and limonene) were obtained from Tokyo Chemical Industry (Shanghai) Development Co., Ltd., Shanghai, China. Aliquots of 0.5 μL of the essential oil (diluted with acetone at five different concentrations) were applied topically to the dorsal thorax of the insects (10 insects per replicate, five replicates per dose). Insects treated with acetone alone were used as controls. Both treated and control insects were then transferred to glass vials (10 insects per vial) and kept in incubators. Insect mortality was checked after 24 h, and the LD_50_ values were calculated using Probit analysis [45]. Positive control, pyrethrins (pyrethrin I and II, 37%) were purchased from Dr. Ehrenstorfer GmbH.

The contact toxicity of the essential oil and the individual compounds against *L. bostrychophila* was tested as described [46]. A 5.5 cm diameter filter paper was treated with 300 μL of the solution of the essential oil. The filter paper after being treated with solid glue was placed in a 5.5 cm diameter Petri dish (with a coating of polyterafluoroethylene) and 10 booklice were put on the filter paper. A cover was put and all the Petri dishes were kept in incubators. Acetone was used as a negative control. Five concentrations (diluted with acetone) and five replicates of each concentration were used. Mortality of insects was observed after 24 h. The LD_50_ values were calculated by using Probit analysis [45].

### 3.5. Repellency

The repellent activity of the essential oil and the individual compounds to *T. castaneum* and *L. bostrychophila* was tested using the area preference method [34]. The essential oil was diluted in acetone to different concentrations (78.63, 15.73, 3.15, 0.63, and 0.13 nL/cm^2^) and acetone was used as the control. Filter paper (9 cm in diameter) was cut in half. 500 μL of treatment solution was placed on one half of the filter paper. The other half was treated with 500 μL of acetone. The two halves of filter are allowed to dry for 30 s. The treated side was then joined to the control side by tape and placed in glass Petri dishes (9 cm in diameter). As for the booklice, Petri dishes (with a coating of polyterafluoroethylene) and filter papers were changed to 5.5 cm in diameter and the concentrations of the essential oil used in the experiments were 63.17, 12.63, 2.53, 0.51, and 0.10 nL/cm^2^. The half filter paper was treated with 150 µL of the solution. For both tests, 20 insects were released in the center of each filter paper disk, and a cover was placed over the Petri dish. Five replicates were used. Counts of the insects present on each strip were made after 2 and 4 h. The percent repellency (PR) of essential oil was then calculated using the formula:

PR (%) = [(*N_c_* − *N_t_*)/(*N_c_* + *N_t_*)] × 100

where *N_c_* is the number of insects present in the negative control half and *N_t_* is the number of insects present in the treated half. The averages were then assigned to different classes (0 to V) using the following scale (percentage repellency) [12]. Class, % repellency: 0, >0.01 to <0.1; I, 0.1–20.0; II, 20.1–40.0; III, 40.1–60.0; IV, 60.1–80.0; and V, 80.1–100. Means and standard errors were conducted by Microsoft Excel 2007 for Windows XP. Analysis of variance (ANOVA) and Tukey’s test were conducted by using SPSS 19.0. Percentage was subjected to arcsine square-root transformation before ANOVA and Tukey’s tests. The averages were then assigned to different classes (0 to V) (Table 4). A commercial repellent, DEET (*N*,*N*-diethyl-3-methylbenzamide), was purchased from the National Center of Pesticide Standards (Shenyang, China) and used as a positive control.

## 4. Conclusions

This work indicates that the essential oil of *E. yunnanensis* rhizomes and its four constituents have potential for being developed into natural insecticides/repellents for the control of insects in stored products. However, further studies are needed to focus on the safety of the essential oil for humans and to improve the potency and stability of these potential insecticides/repellents for practical use.
